# Early supported discharge for older adults admitted to hospital with medical complaints: a systematic review and meta-analysis

**DOI:** 10.1186/s12877-022-02967-y

**Published:** 2022-04-08

**Authors:** Susan Williams, Ann-Marie Morrissey, Fiona Steed, Aoife Leahy, Elaine Shanahan, Catherine Peters, Margaret O’Connor, Rose Galvin, Cliona O’Riordan

**Affiliations:** 1 School of Allied Health, Faculty of Education and Health Sciences, Ageing Research Centre, Health Research Institute, University of Limerick, Limerick, Ireland; 2 Department of Medicine, University Hospital Limerick, Dooradoyle, Limerick, Ireland; 3 Department of Ageing and Therapeutics, University Hospital Limerick, Dooradoyle, Limerick, Ireland; 4 School of Medicine, Faculty of Education and Health Sciences, University of Limerick, Limerick, Ireland

**Keywords:** Early supported discharge, Older adults, Hospitalised, Systematic review, Medical inpatient

## Abstract

**Introduction:**

Early supported discharge (ESD) aims to link acute and community care, allowing hospital inpatients to return home and continue to receive the necessary input from healthcare professionals that they would otherwise receive in hospital. The concept has shown reduced length of stay and improved functional outcomes in stroke patients. This systematic review aims to explore the totality of evidence for the use of early supported discharge in older adults hospitalised with medical complaints.

**Methods:**

A literature search of CINAHL in EBSCO, Cochrane Central Register of Controlled Trials in the Cochrane Library (CENTRAL), EMBASE and MEDLINE in EBSCO was carried out. Randomised controlled trials or quasi-randomised controlled trials were included. The Cochrane Risk of Bias Tool 2.0 was used for quality assessment. The primary outcome measure was hospital length of stay. Secondary outcomes included mortality, function, health related quality of life, hospital readmissions, long-term care admissions and cognition. A pooled meta-analysis was conducted using RevMan software 5.4.1.

**Results:**

Five studies met the inclusion criteria. All studies were of some concern in terms of their risk of bias. Statistically significant effects favouring ESD interventions were only seen in terms of length of stay (REM, MD = -6.04, 95% CI -9.76 to -2.32, I^2^ = 90%, *P* = 0.001). No statistically significant effects favouring ESD interventions were established in secondary outcomes.

**Conclusion:**

ESD interventions can have a statistically significant impact on the length of stay of older adults admitted to hospital for medical reasons. There is a need for further higher quality research in the area, with standardised interventions and outcome measures used.

**Supplementary Information:**

The online version contains supplementary material available at 10.1186/s12877-022-02967-y.

## Introduction

Approximately half of older adults who present to the emergency department (ED) are admitted for inpatient care [1, 2]. Older adults admitted to hospital are more likely to experience reduced mobility and an increased risk of hospital acquired infections which can further extend their hospital length of stay (LoS) [3]. With an increased LoS, older adults are more likely to experience adverse events such as hospital-acquired infection, falls and increased mortality [4].

Early supported discharge (ESD) is a discharge intervention aimed at linking inpatient care and community services to allow patients to return home more than would be otherwise possible with community care, by receiving additional input from healthcare professionals [5]. ESD provided to acute stroke patients has been widely researched. Langhorne and Baylan [5] carried out a Cochrane review of 17 randomised control trials (RCT’s) providing ESD interventions in acute stroke care. An average reduction of six days (MD = ‐5.5; 95% CI ‐3 to ‐8 days; *P* < 0.0001) in hospital LoS was found in the intervention group and reduced admissions to long term care facilities (OR 0.75, 95% CI 0.59 to 0.96, *P* = 0.02). No clear differences were seen in terms of activities of daily living (ADL’s) or patients’ subjective health status or mood. ESD has also been explored in a respiratory population in a feasibility study by Collins, Eneje [6]. In this review, the ESD intervention was provided to patients deemed to be of moderate disability. Typically, ESD teams had approximately 3.1 Whole Time Equivalent (WTE) staff. The composition of staff varied across studies but broadly comprised—medical 0.1 WTE, nursing (range from 0 to 1.2 WTE), physiotherapy 1 WTE, occupational therapy 1 WTE, speech and language therapy 0.3 WTE, therapy assistant 0.4 WTE. Variable levels of social work (range 0 to 0.5 WTE) and secretarial support were also available. Team input began on day one post discharge, with four to five therapy sessions provided weekly for a maximum of three months.

ESD and its’ effect on patient and process outcomes in an older adult population admitted to hospital with medical complaints has been the focus of more recent RCT’s. Parsons, Parsons [7] reported an average reduction in LoS by six days (95% CI 0.6 to 11.3) in older adults who received ESD versus the control group who received usual care. The intervention led by a consultant geriatrician, involved services provided by nursing staff, health care assistants and allied healthcare professionals. Re-admission days spent in hospital in the following six months were also reduced in the ESD group (mean 7.1 days, SD = 12.8 days) when compared to the control group (mean 12.5 days, SD = 24.2 days).

In their review of ESD in acute stroke care, Langhorne and Baylan [5] reviewed carer outcomes (mood, carer satisfaction and subjective health status) but the role of carers in assisting with ESD programmes was not explicitly noted. A systematic review and meta-analysis of 15 RCT’s by Rodakowski, Rocco [8] demonstrated that involving caregivers in the discharge process can reduce hospital readmission risk by up to 24% 180 days post-discharge. In a qualitative study by Georgiadis and Corrigan [9] exploring patient and caregiver experiences of discharge from an acute hospital, both patients and their caregivers were more likely to report negative experiences if they had limited involvement in the discharge process. Patients also report that they felt their concerns of premature discharge were not listened to. Given the positive outcomes of involving caregivers in the discharge process for older adults, ESD would facilitate caregiver involvement to allow for a shared decision-making process between the patient, their caregiver(s), and the healthcare team.

While the evidence to date demonstrates strong support for ESD as an intervention for stroke patients, the totality of evidence regarding the effectiveness of an ESD intervention for older adults admitted to hospital for medical reasons has not been explored. The aim of this systematic review and meta-analysis is to explore the effectiveness of ESD on process and clinical outcomes in older adults admitted to hospital with medical complaints for medical reasons, and if suitable, perform a meta-analysis.

## Methods

### Study design

A systematic review and meta-analysis was carried out. The Preferred Reporting Items for Systematic Reviews and Meta-Analysis (PRISMA) guidelines were followed, see Additional File 1 [10]. The Cochrane Handbook for Systematic Reviews of Interventions were adhered to [11]. The protocol for this review was registered with PROSPERO, ID: CRD42021223112 [12].

### Search strategy & selection criteria

Searches were carried out in CINAHL in EBSCO, Cochrane Central Register of Controlled Trials in the Cochrane Library (CENTRAL), EMBASE and MEDLINE in EBSCO on 21^st^ January 2021. The search strategy was comprised of three sections broadly covering the topics of ESD, older adults and study design. Search strategies were based on those carried out by Langhorne and Baylan [5] and Butterworth, Hays [13]. Full search strategies were reported previously Williams, Morrissey [12]. Studies were limited from the year 1997 when the concept of ESD was introduced for stroke care [14, 15].

Inclusion criteria was as follows:

- Population
  Studies were included if > 50% of the study population were older adults (aged > 65 years) who were admitted to the acute care setting for medical reasons.
- Intervention
  Studies were required to provide an ESD intervention, described as interventions aimed to accelerate patient discharge from hospital once medically stable, and providing patients with the necessary input in the community at the same level of intensity and resources they would receive while in the inpatient setting [5].
- Control
  Studies were required to provide active or usual care to the control group as described by study authors.
- Outcomes
  Studies were included if they measured any of the following outcomes—the primary outcome was length of hospital stay. Secondary outcomes included functional abilities (including the Functional Independence Measure (FIM)), quality of life (including the SF-36), cognition (including the Mini Mental State Examination (MMSE)), carer and patient satisfaction, unscheduled hospital readmission (including frequency), nursing home admission, mortality, and cost.
- Study design
  RCT’s (including cluster trials) and quasi-RCT’s were included.

### Study selection

Results from all databases were imported into in a master Endnote library. Duplicates were removed. SW and RG screened studies independently and in duplicate against the inclusion/exclusion criteria in Endnote 20 by title and abstract. Authors were contacted by SW for further information as required.

Studies in the unsure group underwent a full text review by a third author (CO’R). If an agreement could not be met, a fourth author was consulted (A-MM). Four authors (SW, A-MM, CO’R and RG) reviewed all studies in the final relevant group to ensure they met the inclusion criteria.

### Quality assessment

The Cochrane Risk of Bias Tool 2.0 was used to quality assess the included studies [16]. This tool assesses risk of bias across five domains—the randomisation process, deviations from intended interventions, missing outcome data, measurement of the outcome and selection of the reported result. Quality assessment was carried out independently by SW, CO’R, A-MM and RG and then as a group with any discrepancies discussed and decisions made by majority. Study protocols were used where available to guide the quality assessment.

### Data extraction and statistical analysis

Descriptive data (author, year, country, method, population, intervention, control, outcomes measured) were independently extracted by SW. CO’R and A-MM. The authors of included studies were contacted if data were missing.

The Cochrane Review Manager software (RevMan, V.5.4) was used to perform the statistical analysis. If the mean and SD was not available, the IRQ was multiplied by 0.75 and the difference in the range was multiplied by 0.25 [17]. In reporting follow-up data, if outcomes were not reported at the same timepoints post intervention, the timepoints that aligned most closely were used. In studies that assessed the same outcome but used contrasting scales, the treatment effect was determined using the standardised mean difference (SMD). In studies that measured the same outcome using the same scales, the mean difference (MD) was used. For all outcomes, the denominator in each group was considered as the number of participants allocated to that group at baseline.

The I^2^ statistic was used to determine heterogeneity. An I^2^ of greater than 50% was considered substantial, therefore if I^2^ was ≥ 50% a random effects model (REM) was reported. If I^2^ was ≤ 50% a fixed effects model (FEM) was used. If both the fixed effects model and random effects model reported I^2^ ≥ 50%, the most conservative result was used when dissimilar outcomes were obtained [18].

## Results

### General overview

The PRISMA diagram in Additional File 2 summarises the screening and selection of identified studies. Five RCT’s were included in the qualitative synthesis and meta-analysis.

The characteristics of included studies are summarised in Additional File 3.

All interventions were multi-disciplinary team (MDT) led, however the MDT members involved varied per study. Hospital-based medical doctors (registrar or consultant) were a part of three of the studies [7, 19, 20]. Cunliffe, Gladman [21] and Nikolaus, Specht-Leible [22] provided General Practitioner support. Harris, Ashton [19] nominated a case manager with a nursing background for their MDT intervention. Physiotherapists and occupational therapists were included in all intervention MDT’s, with social workers included by Nikolaus, Specht-Leible [22] and Harris, Ashton [19].

Intervention intensity varied by study. Cunliffe, Gladman [21] provided up to four visits seven days a week for up to four weeks, as did Parsons, Parsons [7] except for a period of up to six weeks. Harris, Ashton [19] also provided a seven-day service but did not specify the maximum length of the intervention. On the contrary, Nikolaus, Specht-Leible [22] provided up to two visits two days a week, while Caplan, Coconis [20] did not provide specific details on the intensity of the intervention provided. The control group for all included studies was usual hospital care for older adults, which generally comprised of inpatient rehabilitation followed by community services as appropriate.

Participants follow up varied from 90 days post intervention by Harris, Ashton [19] to 12 months post intervention [21, 22]. Acute hospital LoS, physical functioning and hospital readmissions were the most common outcomes measured.

### Methodological quality

Study quality under the various domains of the tool for each study can be seen in Additional File 4. Overall, all studies were deemed to be of some concern and all studies performed similarly across the five domains assessed.

### Primary outcome

**Fig. 1:**
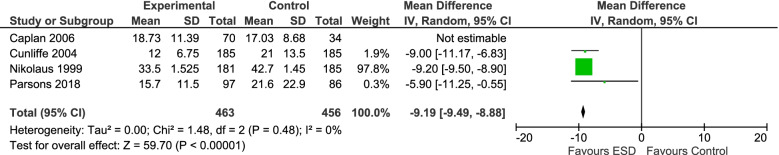
Forest plot for LoS

All included studies except Harris, Ashton [19] measured LoS in terms of days spent as an inpatient during the acute hospital stay. As can be seen in Fig. 1, there were statistically significant effects favouring ESD for LoS between the intervention (*n* = 533) and control groups (*n* = 490), (REM, MD = -6.04, 95% CI -9.76 to -2.32, I^2^ = 90%, *P* = 0.001).

### Secondary outcomes

Function was assessed by all studies, but data were available from three studies [19, 20, 22]. No significant differences were noted in terms of function across the groups (FEM, SMD = 0.08, 95% CI -0.07 to 0.22, I^2^ = 29%, *P* = 0.29), see Additional File 5, supplementary Fig. 1.

Two studies reported health-related quality of life (HRQoL) data suitable for meta-analysis [19, 22]. No statistically significant effects were seen favouring ESD interventions (*n* = 324) versus control groups (*n* = 327), (REM, SMD = 1.71, 95% CI -1.57 to 4.98, I^2^ = 100%, *P* = 0.31), see Additional File 5, supplementary Fig. 2.

Cognition was measured by Caplan, Coconis [20] and Harris, Ashton [19] using the MMSE. No statistically significant effects were seen in the ESD group when compared to the control groups (FEM, MD = -0.47, 95% CI -1.21 to 0.25, I^2^ = 0%, *P* = 0.21), see Additional File 5, Supplementary Fig. 3.

There were no significant differences across the groups in the incidence of admission to long term care (LTC) (FEM, RR 0.89, 95% CI 0.65 to 1.22, I^2^ = 0%, *P* = 0.93) and hospital readmissions (FEM, RR 1.09, 95% CI 0.86 to 1.4, I^2^ = 0%, *P* = 0.39) in the ESD group when compared to the control group, see Additional File 5, supplementary Figs. 4 and 5. All data used for analyses for these outcomes were taken 12 months post intervention.

Mortality was measured by three authors between three- and six-months post intervention [19–21]. There was no difference in risk of mortality between the ESD group (*n* = 398) versus the control group (*n* = 361), (FEM, RR 0.98, 95% CI 0.63 to 1.53, I^2^ = 0%, *P* = 0.93), see Additional File 5, supplementary Fig. 6.

Three studies reported resource costs as an outcome [7, 19, 20]. Harris, Ashton [19] reported their mean cost per patient as NZ$6,524 in the intervention group (*n* = 143) compared to NZ$3,525 in the control group (*n* = 142). On the contrary, Parsons, Parsons [7] reported the average cost per patient per day to be NZ$94 in their intervention group (*n* = 97) and NZ$680 in the control group (*n* = 86). Similarly, Caplan, Coconis [20] reported their mean total cost per patient as A$18,147 in the intervention group (*n* = 70) and A$25,042 in the control group (*n* = 34).

## Discussion

### Statement of key findings

This systematic review examined the impact of ESD interventions in older medical inpatients across a range of clinical and process outcomes. We found a significant reduction in LoS associated with the implementation of ESD among this population. No statistically significant effects favouring ESD interventions were established in mortality, function, HRQoL, hospital readmissions, LTC admissions or cognition.

### Results in context of current literature

Our findings are in-keeping with the primary outcome of reduced LoS reported by Langhorne and Baylan [5] and LTC admissions, as well as reduced risk of adverse outcomes (death) and improvements in participants extended ADL scores and their satisfaction with services when compared to usual care. In terms of care planning post-stroke, a position paper from Miller, Lin [23] reported that ESD interventions enable patients and their caregivers to adapt to their everyday life while still receiving rehabilitation at home.

Zhu, Liu [24] carried out a systematic review and meta-analysis investigating nurse-led discharge planning interventions for inpatients with complex chronic disease or rehabilitation needs. This review of 10 RCT’s (*n* = 3438) found there to be statistically significant reductions in hospital readmissions, readmission LoS and all-cause mortality in those in the intervention groups versus control groups. While the interventions included in this review were not exclusively MDT led as is the case with ESD interventions, they illustrate the positive impacts discharge interventions can have on both process and clinical outcomes as well as the feasibility of ESD interventions being run in practice.

A recent Australian-based observational cohort study by Ramsey, Loveland [25] compared ward based geriatric rehabilitation (*n* = 145) and home-based geriatric rehabilitation (*n* = 18). Those included in the home-based rehabilitation group would have otherwise been kept as inpatients in an acute hospital. They received an MDT based intervention in their homes, which began within 48 h of their hospital discharge. Despite the small number of participants in the home-based group and the data collection for the trial stopping early due to the COVID-19 pandemic, the median physical activity levels were significantly higher (*p* < 0.001) and median sedentary behaviour levels significantly lower (*p* < 0.001) in the home-based group even after matching. None of the studies in our review chose to include physical activity levels as an outcome for their ESD interventions. While physical functioning is commonly assessed in older adults, the addition of physical activity levels may provide a more accurate representation of how older adults are functioning on a day-to-day basis.

In this review, while all interventions were MDT led and based in the participants homes, there were differences in terms of the frequency of the intervention provided, as well as the intensity and timeframe for which it was provided. These inconsistencies as well as the various outcome measures used must be taken into consideration when interpreting results. Heldmann, Werner [26] carried out a systematic review investigating outcome measures that are used in acutely hospitalised older adults. In the 24 studies included in the review, 33 different outcome measures were identified across six categories (functional status, mobility status, hospital outcomes, adverse clinical events, psychological status, and cognitive functioning). The authors reported that outcome measures used showed a large heterogeneity in their matching to the intervention, study sample, and setting, but those that specifically matched the intervention content were more likely to document intervention-induced benefits.

Function was the most commonly assessed clinical outcome in the included studies. The FIM was used to assess function by Caplan, Coconis [20] and Harris, Ashton [19]. In a population of hospitalised older adults, the FIM has been shown to have good construct validity but variable internal consistency across studies [27]. The same authors highlighted low internal consistency with the FIM, as has been shown in previous systematic review by the same authors [28]. In terms of sensitivity to change over time, Wales, Lannin [27] noted moderate-large changes over time for both the FIM and Barthel Index, which was used as a functional assessment by Cunliffe, Gladman [21]. For long term follow-up of those who receive ESD interventions, while the FIM has shown to be both reliable and valid, the sensitivity to change must also be noted as this may influence the accuracy of longer-term results.

### Strengths and weaknesses of the study

Strengths of this systematic review and meta-analysis include the use of PRIMSA guidelines to standardise the conduct and reporting of the study [10]. The high levels of heterogeneity of the studies included was a limitation to this review. Heterogeneity was high clinically in terms of the interventions provided and outcomes measured, with large variation seen across the five included studies. Due to the limited number of relevant studies available for inclusion, it was not possible to conduct a publication bias assessment and/or meta-regression or subgroup analysis.

### Clinical and policy implications

The National Institute for Health and Care Excellence (NICE) published guidelines in 2015 on the transition of adults from hospital to community or care home settings [29]. While these guidelines were targeted to all adults and not just those aged ≥ 65 years, they do recommend a dedicated discharge co-ordinator be assigned to each patient with care needs along with supported discharge including home care and community-based rehabilitation. Of the studies included in this review, Harris, Ashton [19] was the only one to assign a case manager to facilitate discharge for the participants. Specifically focusing on discharging older adults from the New Zealand acute hospital setting to home, Harris, Ashton [19] noted that further research was required to assess the cost-effectiveness of community-based interventions. Similarly, NICE recommended extending research to assess the effectiveness of home interventions post discharge. A systematic review carried out by Zhu, Liu [24] investigating nurse-led discharge programmes for older adults with chronic conditions reported similar findings – an absence of discharge interventions guidelines but the potential for ESD style interventions to reduce LoS and overall costs to the health system. As per this review, discharge interventions such as ESD, have potential to meet the unmet needs of older adults as outlined by NICE.

Comprehensive geriatric assessment (CGA) is the most established model of healthcare for older adults and this is supported by an extensive evidence base [30]. A Cochrane review of 29 studies (*n* = 13,766) carried out in 2017 found that older adults who received CGA in hospital were more likely to be in their own home at a 12 month follow up when compared to usual care (RR 1.06, 95% CI 1.01 to 1.10) [31]. The evidence for cost-effectiveness was inconsistent and imprecise across studies. Furthermore, the review noted that little detail was provided on what interventions are provided after the assessment phase of a CGA. Given that ESD interventions were typically provided by MDT members with specialist geriatric interest and training, it aligns with CGA as a continuation of care. While CGA is not explicitly designed to reduce an older adult’s acute hospital LoS, it can help in keeping older adults at home for longer through identifying a variety of treatable health problems and ultimately lead to better health outcomes. Older adults have reported that continuing to live in their home is influenced by historical, cultural and environmental contexts which shapes their everyday thoughts, activities and what was meaningful for them [32]. ESD can promote independence at home for older adults post hospital admission by allowing them to rehabilitate at home, creating meaningful goals [33]. Given the synergy between the models of care, future research could look to the integration of the two services and their effect on patient, process, and cost outcomes.

### Areas for further research

ESD interventions have the potential to reduce the LoS without adversely affecting clinical outcomes for older adults admitted to hospital for medical reasons. However, further multi-centre larger RCTs are needed that adhere to the CONSORT standardised reporting guidelines [34].

While some research has been carried out focusing on the clinical and process outcomes of ESD interventions in older adults admitted to hospital for medical reasons, little emphasis has been placed on the impact of these interventions on the stakeholders involved including older medical inpatients, their families and carers and healthcare professionals. Neiterman, Wodchis [35] interviewed 17 older adults and their 19 caregivers who had recently been discharged from an acute hospital to home. Those interviewed identified a lack of follow-on care from the acute setting into the community as an area which could have potentially negative implications for patients. In a similar population Georgiadis and Corrigan [9] highlighted the need for families and caregivers to be involved in an older adults discharge plan, with negative experiences of discharge planning linked to a lack of communication and engagement with their healthcare teams. Exploring the impact of ESD interventions on the patient and their caregiver would allow for ESD services to be tailored to include the patient and their caregiver in a shared decision-making process with consent from the older person [36].

## Conclusion

This systematic review and meta-analysis demonstrate that providing ESD intervention to older adults admitted to hospital for acute medical reasons can significantly reduce their acute hospital LoS, without adversely affecting their mortality, function, HRQoL, hospital readmissions, LTC admissions and cognition. Future research should focus on using standardised interventions, outcome measures and establishing the cost–benefit of ESD in this population.

## Supplementary Information

**Additional file 1.** PRISMA Checklist.

**Additional file 2.** PRISMA Flow Diagram.

**Additional file 3.** Characteristics of Included Studies.

**Additional file 4.** Cochrane Risk of Bias Tool 2.0.

**Additional file 5.** Forest Plots for Secondary Outcomes.

## Data Availability

The authors declare that the data supporting the findings of this study are available within the article and its supplementary information files.

## Abbreviations

95% CI: 95% Confidence interval
ADL’s: Activities of daily living
BI: Barthel index
CAM: Confusion assessment method
CGA: Comprehensive geriatric assessment
ED: Emergency department
ESD: Early supported discharge
FEM: Fixed effects model
FIM: Functional independence measure
GDS: Geriatric depression scale
GP: General practitioner
HCA: Healthcare assistant
HRQoL: Health related quality of life
IADL's: Instrumental activities of daily living
IQR: Interquartile range
L: Low risk of bias
LoS: Length of stay
LTC: Long term care
MD: Mean difference
MDT: Multi-disciplinary team
MMSE: Mini mental state examination
NICE: National institute for health and care excellence
OARS: Older americans resources and services instrument
PRISMA: Preferred reporting items for systematic reviews and meta-analysis
RCT: Randomised controlled trial
REM: Random effects model
RR: Risk ratio
SC: Some concerns risk of bias
SF-36: 36 Item short form survey
SMD: Standard mean difference
