# Supplementary material for: Early supported discharge for older adults admitted to hospital with medical complaints: a systematic review and meta-analysis
Source: BMC Geriatr. 2022 Apr 8;22:302. doi: 10.1186/s12877-022-02967-y (PMC8990486; doi:10.1186/s12877-022-02967-y)
Supplement: Supplementary file 2 — Additional file 2. PRISMA Flow Diagram. [file 12877_2022_2967_MOESM2_ESM.docx]

**Additional File 2: PRISMA Flow Diagram**

Studies included in quantitative synthesis (meta-analysis)
(n = 5)

Records excluded
(n = 56,376)

Records screened
(n = 56,418)

Records after duplicates removed
(n = 56,418)

## **Identification**

## **Eligibility**

## **Included**

## **Screening**

Additional records identified through other sources
(n = 0)

Records identified through database searching
(n = 74,771)

Full-text articles assessed for eligibility
(n = 42)

Full-text articles excluded, with reasons (n = 37)

Not an RCT (n = 10)

ED based intervention; participants not admitted for inpatient care (n = 6)

Full text unavailable (n = 5)

Community based intervention (n = 5)

Not ‘early’ discharge planning (n = 4)

No relevant outcomes (n = 4)

Not MDT intervention (n = 2)

Majority orthopaedic population (n = 1)

Studies included in qualitative synthesis
(n = 5)

**Full-text articles excluded, with reasons (n = 37)**

Not an RCT (n = 10)

(1-10)

ED based intervention; participants not admitted for inpatient care (n = 6)

(11-16)

Full text unavailable (n = 5)

(17-21)

Community based intervention (n = 5)

(22-26)

Not ‘early’ discharge planning (n = 4)

(27-30)

No relevant outcomes (n = 4)

(31-34)

Not MDT intervention (n = 2)

(35, 36)

Majority orthopaedic population (n = 1)

(37)

**Reference List**

1. Beauchet O, Launay C, De Decker L, Annweiler C. Mobile geriatric team and length of hospital stay among older inpatients: A case-control pilot study. European Geriatric Medicine. 2013;4:S90.

2. Hough P, Gleeson S, Shkuratova N, Coker F, Williams C. Introducing a clinically effective allied health rapid discharge team within a complex aged subacute in-patient cohort on a cost recovery basis: the Supported Patient centred Early Discharge (SPeED) initiative. Australian Health Review. 2020;44(6):931-4.

3. Lin FOY, Luk JKH, Chan TC, Mok WWY, Chan FHW. Effectiveness of a discharge planning and community support programme in preventing readmission of high-risk older patients. Hong Kong medical journal = Xianggang yi xue za zhi. 2015;21(3):208-16.

4. Mas MÀ, Inzitari M, SabatÉ S, SantaeugÈNia SJ, Miralles R. Hospital-at-home Integrated Care Programme for the management of disabling health crises in older patients: comparison with bed-based Intermediate Care. Age & Ageing. 2017;46(6):925-31.

5. Mas MA, Inzitari M. A critical review of Early Supported Discharge for stroke patients: from evidence to implementation into practice. Int J Stroke. 2015;10(1):7-12.

6. Parker S, Oliver P, Pennington M, Bond J, Jagger C, Enderby P, et al. Rehabilitation of older patients: day hospital compared with rehabilitation at home. A randomised controlled trial. Health Technology Assessment. 2009;13(37):1-168.

7. Watkins L, Hall C, Kring D. Hospital to home: a transition Program for frail older adults. Professional Case Management. 2012;17(3):117-25.

8. Vaid C, Leung S, Costello K. Efficacy of facilitated hospital discharge program (FHDP) on post-hospital outcomes of elderly patients. Journal of the American Geriatrics Society. 2013;61:S217.

9. Stott DJ, Buttery AK, Bowman A, Agnew R, Burrow K, Mitchell SL, et al. Comprehensive geriatric assessment and home-based rehabilitation for elderly people with a history of recurrent non-elective hospital admissions. Age and ageing. 2006;35(5):487-91.

10. Allen J, Hutchinson AM, Brown R, Livingston PM. Quality care outcomes following transitional care interventions for older people from hospital to home: a systematic review. BMC Health Services Research. 2014;14(1):346-.

11. Caplan GA, Williams AJ, Daly B, Abraham K. A randomized, controlled trial of comprehensive geriatric assessment and multidisciplinary intervention after discharge of elderly from the emergency department--the DEED II study. Journal of the American Geriatrics Society. 2004;52(9):1417‐23.

12. Ong MK, Jones L, Aoki W, Belin TR, Bromley E, Chung B, et al. A Community-Partnered, Participatory, Cluster-Randomized Study of Depression Care Quality Improvement: Three-Year Outcomes. Psychiatric Services. 2017;68(12):1262-70.

13. O'Riordan Y, Bernard P, Maloney P, Enright A, McGrath C. Safer transitioning Optimising Frail Elderly Patients Care From Hospital to Home. International Journal of Integrated Care (IJIC). 2017;17:1-2.

14. Pedersen LH, Gregersen M, Barat I, Damsgaard EM. Early geriatric follow-up after discharge reduces readmissions – A quasi-randomised controlled trial. European Geriatric Medicine. 2016;7(5):443-8.

15. Pedersen LH, Gregersen M, Barat I, Damsgaard EM. Early geriatric follow-up visits to nursing home residents reduce the number of readmissions: a quasi-randomised controlled trial. European geriatric medicine. 2018;9(3):329‐37.

16. Ebrahimi Z, Eklund K, Dahlin-Ivanoff S, Jakobsson A, Wilhelmson K. Effects of a continuum of care intervention on frail elders' self-rated health, experiences of security/safety and symptoms: a randomised controlled trial. Nordic journal of nursing research. 2017;37(1):33‐43.

17. Sejr Kirring L, Gregersen M, Damsgaard EM. Can readmissions be reduced by a hospital-at-home multidisciplinary geriatric team? European Geriatric Medicine. 2013;4:S170.

18. Shahla S, Gregersen M, Damsgaard EM. Hospital-at-home by a multidisciplinary geriatric team reduces mortality after discharge from an emergency medical department. European Geriatric Medicine. 2013;4:S173.

19. Cunliffe AL, Dewey M, Gladman JRF, Harwood RH, Husbands SL, Miller PS. Evaluation of an early discharge scheme for elderly people: outcomes at 3 months. <Http://wwwnottinghamacuk/rehab/whatwentright/eds_gladmanpdf>. 2003.

20. Cunliffe AL, Gladman JRF, Husbands SL, Miller P, Dewey ME, Harwood RH. Sooner and healthier: a randomised controlled trial and interview study of an early discharge rehabilitation service for older people. Age & Ageing. 2004;33(3):246-52.

21. Cunliffe AL, Dewey ME, Gladman JRF, Harwood RH, Husbands SL, Miller PS. Evaluation of an early discharge scheme for older people: outcomes at 12 months. <Http://wwwnottinghamacuk/rehab/whatwentright/eds_gladmanpdf>. 2003.

22. Shepperd S, Harwood D, Jenkinson C, Gray A, Vessey M, Morgan P. Randomised controlled trial comparing hospital at home care with inpatient hospital care. I: three month follow up of health outcomes. BMJ (Clinical research ed). 1998;316(7147):1786‐91.

23. Shepperd S, Harwood D, Gray A, Vessey M, Morgan P. Randomised controlled trial comparing hospital at home care with inpatient hospital care. II: cost minimisation analysis. BMJ (Clinical research ed). 1998;316(7147):1791-6.

24. Parker SG, Oliver P, Pennington M, Bond J, Jagger C, Enderby P, et al. Rehabilitation of older patients: day hospital compared with rehabilitation at home. Clinical outcomes. Age & Ageing. 2011;40(5):557-62.

25. Béland F, Bergman H, Lebel P, Dallaire L, Fletcher J, Contandriopoulos A-P, et al. Integrated services for frail elders (SIPA): a trial of a model for Canada. Canadian journal on aging = La revue canadienne du vieillissement. 2006;25(1):5-42.

26. Bernabei R, Landi F, Gambassi G, Sgadari A, Zuccala G, Mor V, et al. Randomised trial of impact of model of integrated care and case management for older people living in the community. BMJ (Clinical research ed). 1998;316(7141):1348‐51.

27. Altfeld SJ, Shier GE, Rooney M, Johnson TJ, Golden RL, Karavolos K, et al. Effects of an enhanced discharge planning intervention for hospitalized older adults: a randomized trial. The Gerontologist. 2013;53(3):430-40.

28. Avlund K, Jepsen E, Vass M, Lundemark H. Effects of comprehensive follow-up home visits after hospitalization on functional ability and readmissions among old patients. A randomized controlled study. Scandinavian Journal of Occupational Therapy. 2002;9(1):17-22.

29. Buurman BM, Parlevliet JL, Allore HG, Blok W, van Deelen BAJ, van Charante EPM, et al. Comprehensive geriatric assessment and transitional care in acutely hospitalized patients: the transitional care bridge randomized clinical trial. JAMA internal medicine. 2016;176(3):302-9.

30. Nikolaus T, Bach M. Preventing falls in community-dwelling frail older people using a home intervention team (HIT): results from the randomized Falls-HIT trial. Journal of the American Geriatrics Society. 2003;51(3):300‐5.

31. Toye C, Parsons R, Slatyer S, Aoun SM, Moorin R, Osseiran-Moisson R, et al. Outcomes for family carers of a nurse-delivered hospital discharge intervention for older people (the Further Enabling Care at Home Program): Single blind randomised controlled trial. International Journal of Nursing Studies. 2016;64:32-41.

32. Turunen KM, Aaltonen-Määttä L, Törmäkangas T, Rantalainen T, Portegijs E, Keikkala S, et al. Effects of an individually targeted multicomponent counseling and home-based rehabilitation program on physical activity and mobility in community-dwelling older people after discharge from hospital: a randomized controlled trial. Clinical Rehabilitation. 2020;34(4):491-503.

33. Parsons M, Senior H, Kerse N, Mei-hua C, Jacobs S, Anderson C. Randomised trial of restorative home care for frail older people in New Zealand. Nursing Older People. 2017;29(7):27-33.

34. Provencher V, Clemson L, Wales K, Cameron ID, Gitlin LN, Grenier A, et al. Supporting at-risk older adults transitioning from hospital to home: who benefits from an evidence-based patient-centered discharge planning intervention? Post-hoc analysis from a randomized trial. BMC geriatrics. 2020;20(1):84.

35. Aoun SM, Stegmann R, Slatyer S, Hill KD, Parsons R, Moorin R, et al. Hospital postdischarge intervention trialled with family caregivers of older people in Western Australia: Potential translation into practice. BMJ Open. 2018;8(11).

36. Clemson L, Lannin NA, Wales K, Salkeld G, Rubenstein L, Gitlin L, et al. Occupational Therapy Predischarge Home Visits in Acute Hospital Care: a Randomized Trial. Journal of the American Geriatrics Society. 2016;64(10):2019‐26.

37. Richards SH, Coast J, Gunnell DJ, Peters TJ, Pounsford J, Darlow MA. Randomised controlled trial comparing effectiveness and acceptability of an early discharge, hospital at home scheme with acute hospital care. BMJ (Clinical research ed). 1998;316(7147):1796‐801.
