# Supplementary material for: Early supported discharge for older adults admitted to hospital with medical complaints: a systematic review and meta-analysis
Source: BMC Geriatr. 2022 Apr 8;22:302. doi: 10.1186/s12877-022-02967-y (PMC8990486; doi:10.1186/s12877-022-02967-y)
Supplement: Supplementary file 3 — Additional file 3. Characteristics of Included Studies. [file 12877_2022_2967_MOESM3_ESM.docx]

**Additional File 3: Characteristics of Included Studies**

| **Author** | **Year** | **Country** | **Methods** | **Population** | **Intervention** | **Control** | **Outcomes Measured** |
| --- | --- | --- | --- | --- | --- | --- | --- |
| Caplan et al. | 2006 | Australia | RCT, randomisation by computer generated random numbers coded into opaque envelopes by a secretary using a 2:1 distribution.  Assessors were blinded on initial assessment but unblinded on subsequent assessments. | Geriatric inpatients referred for geriatric rehabilitation with a LoS greater than six days in the Prince of Wales Hospital, Sydney, Australia between April 2000 and October 2002. Inpatients must have been suitable for rehabilitation and lived in the local hospital area (not nursing home residents). 812 patients were referred for geriatric rehabilitation, 568 did not meet inclusion criteria, 140 declined to participate.  104 participants randomised - mean age intervention 83.86 (SD 7.8), mean age control 84 (SD 7.02); FIM at enrolment intervention 75.46 (SD 22.1), FIM at enrolment control 78.47 (SD 19.13). | Home rehabilitation service (n = 70) Patients kept in hospital until they could independently transfer and mobilise independently to the toilet. Home rehabilitation provided by an outreach MDT (who also provide home rehabilitation for orthogeriatrics and admission avoidance Hospital at Home service). MDT composed of nurses, physiotherapists, occupational therapists and doctors. Patients on average visited a mean of 20 times during the intervention. Equipment required was provided for free for up to three months. | In hospital rehabilitation ward (n = 34) Patients transferred to a geriatric rehabilitation ward once a bed was available, and their acute illness was settling. | Primary -  Incidence of delirium measured by the CAM - assessed every second day from enrolment to discharge (during acute phase and rehabilitation) Secondary - FIM MMSE GDS  assessed on discharge from rehabilitation, one and six month follow up Satisfaction survey within one week of discharge from rehabilitation for the patients, carers and GP's. Acute LoS Rehabilitation LoS Total length of episode of care from admission to end of rehabilitation time Hospital bed days Odds ratio for delirium in home rehabilitation group during rehabilitation phase Costs - acute phase, rehabilitation phase, total Re-admissions within 28 days of ending rehabilitation Mortality to six months |

| Cunliffe et al. | 2004 | UK | RCT, computer generated balanced randomisation within strata. Stratification based on diagnostic group (stroke, fracture or other medical/surgical condition) and BI (≤ 14/20 or ≥ 14/20). Assessors blinded to allocation at all assessment stages. | Patients aged ≥65 years residing in the Nottingham Health Authority boundary, medically fit for discharge and have rehabilitation needs that could be met with a home-based rehabilitation programme.  Patients requiring constant/overnight care excluded. Median age intervention 80 years (IRQ 73-85). mean age control 79 years (IRQ 72-86); median preadmission BI intervention 18 (IRQ 17-20), median pre admission BI control 18 (IRQ 17-20). | Early Discharge and Rehabilitation Service (EDRS) (n = 185) Multi-disciplinary team staffed by two occupational therapists, two physiotherapists, three nurses, a community care officer who liaised with social services, seven rehabilitation assistants and secretarial support.  No doctors on the EDRS, medical care given by primary team while inpatient and GP when at home.  ERDS visits could be for the purpose of assessment or monitoring. Patients seen by the EDRS as soon as possible after hospital discharge.  Visits provided up to four times a day, up to seven days/week between 8:00 and 22:00. Intervention provided for up to four weeks (as per individual patient needs). Standard after-care services also available as required. | Usual hospital care (n = 185) Inpatient management until fit for home, using existing after-care services (hospital outpatient department rehabilitation, geriatric day hospitals, usual social services) as required. | LoS Hospital bed days used from randomisation to three months and 12 months Hospital outpatient visits over 12 months GP visits over 12 months Readmissions within three and 12 months Long term care admissions by three and 12 months Geriatric day hospital attendances over 12 months Social care services provided over 12 months Health outcomes recorded at three and 12 months - Survival Place of residence BI Nottingham Extended ADL score General Health Questionnaire 12 item version - patients and carers Euroqol EQ-5D Interviews before hospital discharge, four weeks and three months after discharge for patients Interviews six-eight weeks after the care episode for ERDS staff |
| --- | --- | --- | --- | --- | --- | --- | --- |
| Harris et al. | 2005 | New Zealand | RCT, randomisation by a computer  generated randomisation service independent of all parties. Assessors not involved in patient care but not blinded at any assessment stage. | Hospital inpatients aged ≥55 years admitted to an acute hospital in central Auckland for medical reasons. Admission Prevention Group - presented to hospital with a medical issue but who could be managed at home with admission by the hospital at home programme (and nothing else). They must have been in the ED or Acute Assessment Area less than 36 hours and not admitted to an inpatient ward. Early Discharge Group - admitted to hospital ward for medical care but with the support of the hospital at home service could be discharged home earlier than otherwise would have been possible. Patients excluded if booked for major surgery within 36 days of randomisation or did not have suitable living arrangements.  841 referrals (294 admission prevention, 547 early discharge) - 285 (33.6%) randomised, 556 not randomised (52.8% not eligible, 13.6% not consented).  Mean age 80 years (80.4% ≥75 years, 3.9% ≤65 years); mean baseline MMSE hospital at home group 27.2 (SD 2.8), mean baseline MMSE hospital group 26.5 (SD 3.6); mean baseline FIM hospital at home group 100.5 (SD 13.8), mean baseline FIM hospital at home group 98.6 (SD 13.6). | Early discharge (n = 143) Nursing led MDT (hospital at home registrar, consultant geriatricians, occupational therapy, physiotherapy, social work) co-ordinating patient care in their own home. Patient care planning operated as an outreach programme.  Seven days/week, 10 hours/day nursing availability.  24 medical on call by a geriatrician. Daily nursing review and adjustment of care plan. Up to 24 hour live in home carer. MDT providing rehabilitation in the home.  Discharge handover to ongoing support services. | Usual hospital care (n = 142) | Primary -  FIM MMSE Modified OARS assessment Secondary -  Self-reported recovery SF-36 (acute form) at 90 days after randomisation Withdrawal from the study Hospital readmissions Falls Bowel problems Bladder problems Confusion Admission to institution for permanent care Death Acceptability survey for patients and caregiver at 90 days Carer Strain Index Resource costs 30 days following randomisation - in-hospital services, hospital at home, community services Perceived changes in personal costs |
| Nikolaus et al. | 1999 | Germany | RCT, randomisation by sealed envelopes containing group assignments using a random number sequence. Assessors blinded at initial assessment but not for follow up assessments. | Patients aged ≥65 years with acute disease referred to the geriatric centre at the University Hospital of Heidelberg. Patients who lived at home prior to admission, had multiple chronic conditions or functional deterioration after convalescence or were at risk of nursing home placement.  Patients excluded if they had a terminal illness or severe dementia or lived ≥15km from the home intervention team base. Mean age 81.4; mean BI 71 | CGA and in hospital and post discharge follow-up treatment by an interdisciplinary home intervention team (n = 181). Home intervention team consisted of three nurses, a physiotherapist, an occupational therapist, a social worker, and a secretary. The team worked closely with hospital staff and the primary care physician.  While an inpatient, the patient received additional treatment and one home visit was carried out during the inpatient stay to evaluate the patient's home environment and provide aids as required.  After discharge from hospital to home, treatment was provided which home services could not or could not immediately provide for as long as necessary. The first home visit was carried out within three days of discharge.  Treatment provided by the physiotherapist and occupational therapist (by the Bobath concept) was provided twice a week, up to twice a day, for a minimum of 30 minutes). | CGA with recommendations followed by usual care at home (n = 179).  Assessment of ADL's and cognition followed by usual care in hospital and at home (n = 185). | LoS Functional Status Discharge Destination Survival Readmissions Nursing home placement Direct costs At 12 month follow up -  Number of medications Self-perceived health score Life satisfaction score ADL score Living location Re-hospitalisations Nursing home placement Visits to physicians Use of community services Direct costs for patients |
| Parsons et al. | 2018 | New Zealand | RCT, randomisation by computer generated randomisation sequence. Assessors blinded to all assessments. | Inpatients aged ≥65 years in Waikato, New Zealand. Included patients must not have needed ongoing acute hospital based treatment as per the consultant geriatrician, consented to treatment at home and agreed with objectives set by the referring inter-disciplinary team. Included patients must have been deemed to have potential for partial or complete recovery with home rehabilitation within six weeks, able to stand and transfer with assistance of one, the patient was at borderline level of function post a recent acute illness/injury with an associated reduction in ADL's and/or IADL's who without input from the team were considered likely to return to full functional recovery or likely to fail to manage satisfactory at home despite conventional community support and therefore deemed to be at risk of readmission or institutionalisation. Mean age intervention 79.8 (SD 7.2), mean age control 78.7 (SD 8.2) | Early supported discharge team (START) (n = 97) HCA's, registered nurses, physiotherapy and occupational therapy, consultant geriatricians providing weekly input through case conferencing. HCA's provided up to four visits/day, seven days/week utilising functional rehabilitation principles for a maximum of six weeks (exceptions made to allow patients to reach maximum potential recovery). Goal setting encouraged to help support development of a care plan. Once at home, direct clinical care responsibility is carried out by the GP. | Usual care (n = 86) Discharge planning from the hospital to the patient's place of residence and subsequent community-based services as required. | Primary - LoS Secondary -  Cumulative LoS in the six months following randomisation (re-admissions) Health related costs interRAI-CA ADL self-performance scale Instrumental ADL Capacity scale |
