# Supplementary material for: Early supported discharge for older adults admitted to hospital with medical complaints: a systematic review and meta-analysis
Source: BMC Geriatr. 2022 Apr 8;22:302. doi: 10.1186/s12877-022-02967-y (PMC8990486; doi:10.1186/s12877-022-02967-y)
Supplement: Supplementary file 4 — Additional file 4. Cochrane Risk of Bias Tool 2.0. [file 12877_2022_2967_MOESM4_ESM.docx]

**Additional File 4: Cochrane Risk of Bias Tool 2.0**

| **Author** | **Year** | **Domain 1** | **Domain 2** | **Domain 3** | **Domain 4** | **Domain 5** | **Overall** |
| --- | --- | --- | --- | --- | --- | --- | --- |
| Caplan | 2006 | L | SC | L | L | SC | SC |
| Cunliffe | 2004 | L | SC | L | L | SC | SC |
| Harris | 2005 | L | SC | L | L | SC | SC |
| Nikolaus | 1999 | L | L | L | L | SC | SC |
| Parsons | 2018 | L | SC | L | SC | L | SC |

**Key:**

| Domain 1 | Risk of bias arising from randomisation process |
| --- | --- |
| Domain 2 | Risk of bias due to deviations from the intended interventions |
| Domain 3 | Missing outcome data |
| Domain 4 | Risk of bias in measurement of the outcome |
| Domain 5 | Risk of bias in the selection of the reported result |
| Overall | Overall risk of bias |
