# Supplementary material for: Early supported discharge for older adults admitted to hospital with medical complaints: a systematic review and meta-analysis
Source: BMC Geriatr. 2022 Apr 8;22:302. doi: 10.1186/s12877-022-02967-y (PMC8990486; doi:10.1186/s12877-022-02967-y)
Supplement: Supplementary file 5 — Additional file 5. Forest Plots for Secondary Outcomes. [file 12877_2022_2967_MOESM5_ESM.docx]

**Additional File 5: Forest Plots for Secondary Outcomes**

*
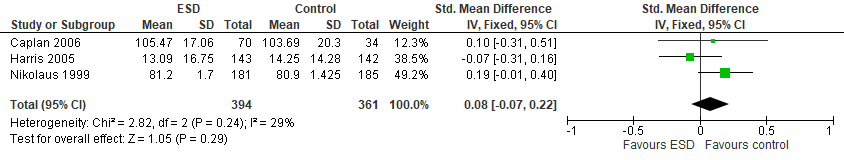
*

*Supplementary figure 1: Forest plot for function*

*
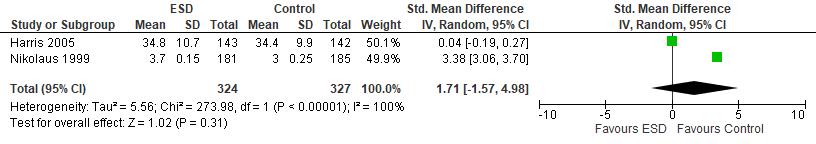
*

*Supplementary figure 2: Forest plot for HRQoL*

*
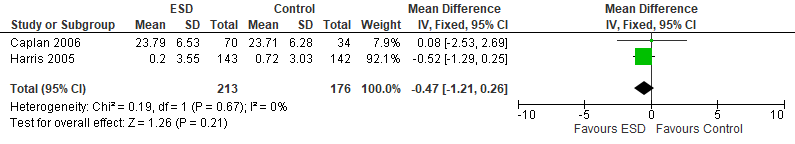
*

*Supplementary figure 3: Forest plot for cognition*

*
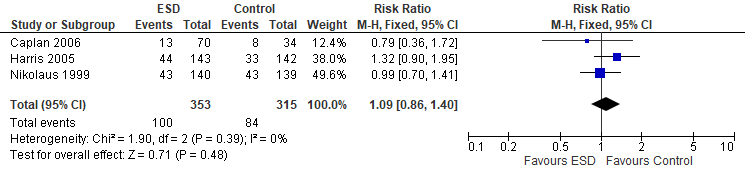
*

*Supplementary figure 4: Forest plot for hospital re-admissions*

*
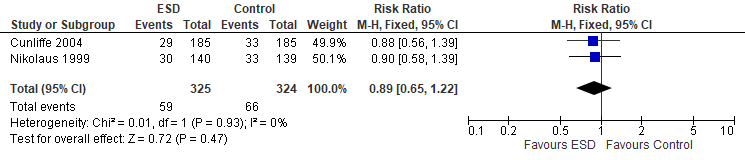
*

*Supplementary figure 5: Forest plot for LTC admissions*

*
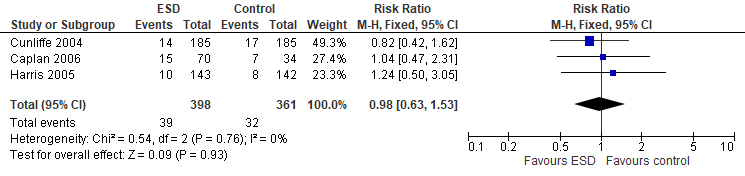
*

*Supplementary figure 6: Forest plot for mortality*
